# Therapeutic Impact of Dobutamine Stress Echocardiography in Chronic Coronary Syndrome: A Real-World Monocentric Study in a Moroccan Population

**DOI:** 10.7759/cureus.99512

**Published:** 2025-12-17

**Authors:** Ghita Bennis, Sara Hafid, Mohamed Samy Lebbar, Youssef Daoudi, Malak Alaoui Yazidi, Oualid Mohammed Taibi, Hajar Rabii, Jamal Kheyi, Fatimazahra Merzouk, Ghali Benouna

**Affiliations:** 1 Department of Cardiology, Cheikh Khalifa International University Hospital, Mohammed VI University of Health Sciences (UM6SS), Casablanca, MAR; 2 Department of Cardiology, Mohammed VI International University Hospital, Mohammed VI University of Health Sciences (UM6SS), Casablanca, MAR; 3 Intensive Care and Arrhythmia Unit, Mohammed V Military Teaching Hospital, Faculty of Medicine and Pharmacy, Mohammed V University, Rabat, MAR

**Keywords:** coronarography, coronary revascularization, dobutamine stress echocardiography, morocco, pretest probability

## Abstract

Background and objective

Dobutamine stress echocardiography (DSE) is a commonly used, radiation-free method for identifying inducible ischemia in patients with chronic coronary syndromes (CCSs). Contemporary European guidelines promote ischemia-guided management and recommend functional imaging in patients with an intermediate-to-high likelihood of obstructive coronary artery disease (CAD); however, real-world data on the therapeutic impact of DSE, particularly in high-risk metabolic populations, remain limited. The objective of this study was to evaluate the correlation between DSE findings and myocardial revascularization in patients with significant CAD.

Methods

We conducted a retrospective, single-center study in the cardiology department of a Moroccan university hospital. All patients undergoing DSE between November 2024 and November 2025 were screened (n = 133); those with a positive or equivocal DSE who subsequently underwent coronary angiography formed the study cohort (n = 38). Significant CAD was defined as ≥50% left main stenosis or ≥70% stenosis of a major epicardial vessel. We assessed the prevalence of significant CAD, patterns of revascularization (medical therapy, percutaneous coronary intervention (PCI), and coronary artery bypass grafting (CABG)), the relationship between DSE-derived ischemic burden and coronary anatomy, and the performance of the 2024 ESC pretest probability (PTP) categories.

Results

Significant CAD was present in 30/38 patients (78.9%), and all CAD-positive patients underwent revascularization (25 PCI and five CABG). PCI was used exclusively in single-vessel disease, whereas CABG was preferred in multivessel or left main disease. The mean number of ischemic segments was higher in CAD-positive than in CAD-negative patients (3.64 vs. 1.50; p = 0.0044), and territorial concordance between DSE and angiography was 67.6%. The correlation between the number of ischemic segments and the number of diseased vessels was modest (r = 0.19). The ESC 2024 PTP strata did not discriminate between CAD-positive and CAD-negative patients; notably, most patients with low or very low PTP and significant CAD were diabetic.

Conclusions

In this real-world Moroccan cohort, DSE demonstrated a high diagnostic and therapeutic yield, with ischemic burden and localization strongly influencing revascularization strategy. These findings underscore the limitations of PTP-based approaches in high-risk metabolic populations and support DSE as a practical gatekeeper to invasive angiography and guideline-based revascularization in CCS.

## Introduction

Dobutamine stress echocardiography (DSE) is a widely available, radiation-free imaging modality used to detect inducible ischemia in chronic coronary syndrome (CCS). The 2024 European Society of Cardiology (ESC) guidelines recommend functional imaging in patients with an intermediate-to-high clinical likelihood of obstructive coronary artery disease (CAD), emphasizing ischemia-guided rather than anatomy-only decision-making [1]. Despite its widespread global use, DSE remains understudied in North Africa, limiting understanding of its correlation with myocardial revascularization in local patient populations.

Contemporary ESC/European Association for Cardio-Thoracic Surgery (EACTS) revascularization guidelines support percutaneous coronary intervention (PCI) for most single-vessel or less complex disease and coronary artery bypass grafting (CABG) for left main or multivessel CAD, especially in patients with diabetes or impaired left ventricular function [2]. Observational studies have consistently shown that patients with a larger ischemic burden derive greater benefit from revascularization than from medical therapy alone [3].

Within this framework, DSE serves not only as a diagnostic test but also as a key tool for selecting patients for invasive angiography and informing therapeutic strategy.

The aim of this study was to assess how DSE-derived ischemic burden relates to coronary anatomy and subsequent revascularization decisions (medical therapy, PCI, or CABG) in a real-world cohort of patients with CCS referred for coronary angiography after a positive or equivocal DSE.

## Materials and methods

Study design and population

This was a retrospective, single-center study conducted in the cardiology department of the Cheikh Khalifa International University Hospital, Mohammed VI Foundation of Sciences and Health, Casablanca, Morocco. All consecutive patients who underwent DSE between November 2024 and November 2025 were screened (n = 133).

Of these, 93 patients completed a full DSE protocol with analyzable imaging and clinical data. According to institutional practice, only patients with a positive or equivocal DSE were referred for coronary angiography, whereas negative tests were not explored invasively. Ultimately, 38 patients with a positive or equivocal DSE who underwent coronary angiography constituted the study population. This selection process enabled the evaluation of predictive and therapeutic implications of DSE but did not allow assessment of specificity or negative predictive value.

Patients were eligible for inclusion if they were aged 18 years or older and had completed a full, analyzable DSE protocol. Only individuals who demonstrated a positive or equivocal DSE and who subsequently underwent coronary angiography during the same diagnostic episode, in accordance with institutional practice, were retained for analysis.

Examinations were excluded when the stress test was submaximal, defined as a failure to achieve at least 85% of the age-predicted maximal heart rate, or when the images were noninterpretable because of baseline or stress-induced artifacts that masked wall-motion assessment. DSE procedures performed solely for myocardial viability rather than ischemia assessment were also excluded, as were cases with incomplete imaging loops or missing essential clinical or angiographic data. Finally, coronary angiographies performed for indications unrelated to DSE interpretation, such as preoperative evaluation, were not considered in the study.

DSE protocol

All examinations were performed using a Philips Affiniti ultrasound system (Philips Healthcare, Bothell, Washington, USA) equipped with an S5-1 sector probe. The standard incremental dobutamine protocol was applied (10, 20, 30, 40 μg/kg/min), with atropine administered when required to achieve the target heart rate.

Blood pressure, heart rate, and continuous 12-lead ECG monitoring were recorded throughout the procedure. Echocardiographic images were obtained at rest, during each infusion stage, and at peak stress.

Criteria for a positive test

A DSE was considered positive when it demonstrated new or worsening regional wall-motion abnormalities involving at least three of the 17 myocardial segments. The test was also classified as positive if typical angina occurred during stress in conjunction with ECG changes suggestive of ischemia, including ≥1 mm ST-segment depression, ST elevation, or dynamic T-wave inversion. Stress-induced ventricular arrhythmias, such as polymorphic premature ventricular contractions or episodes of ventricular tachycardia, were likewise interpreted as indicative of a positive result. In addition, incomplete examinations that raised a reasonable suspicion of inducible ischemia were labelled as equivocal and, for diagnostic purposes, were analyzed together with positive tests.

Coronary angiography analysis

CAD was classified as significant when angiography demonstrated a stenosis of at least 50% in the left main coronary artery or 70% or more in any major epicardial vessel.

Collected variables

Data were collected from medical records, including demographic characteristics (age, sex, and weight) and major cardiovascular risk factors such as high blood pressure, diabetes, dyslipidemia, smoking, and chronic kidney disease. Presenting symptoms were also documented, distinguishing between typical angina, atypical chest pain, dyspnea, and palpitations. For each patient, the pretest probability (PTP) of CAD was calculated in accordance with the 2024 ESC guidelines. Electrocardiographic results under dobutamine (positive or negative) and stress echocardiography findings, including test positivity or equivocality and the number of ischemic segments, were systematically collected. Finally, angiographic data included the presence or absence of significant coronary lesions and the number of vessels involved.

All medical records were fully de-identified prior to data extraction and analysis, in accordance with institutional confidentiality and ethical standards.

Statistical analysis

Statistical analyses were performed using standard methods. Continuous variables are presented as mean ± standard deviation and were compared between groups using Student’s t-test. Categorical variables are reported as counts and percentages and were compared using Fisher’s exact test, given the small sample size and the presence of expected cell counts <5 in several contingency tables.

For PTP, ESC 2024 categories were collapsed into two groups (very low/low vs. moderate/high) for comparative analysis. The relationship between the number of ischemic segments on DSE and the number of significantly diseased coronary vessels was evaluated using Pearson’s correlation coefficient.

A p-value <0.05 was considered statistically significant for group comparisons. The correlation analysis was reported descriptively and was not intended for formal hypothesis testing.

## Results

Baseline characteristics

A total of 38 patients underwent coronary angiography after a positive or equivocal DSE. Significant CAD was confirmed in 30 patients (78.9%), while eight patients (21.1%) had no significant stenosis.

Table 1 summarizes baseline characteristics of the overall cohort according to angiographic status.

**Table 1:** Baseline characteristics of the 38 patients undergoing coronary angiography after dobutamine stress echography Quantitative variables were compared using the Mann-Whitney U test (U reported), and qualitative variables using Fisher’s exact test with ORs and 95% CIs. CAD: coronary artery disease; CAD+: significant coronary artery disease; CAD-: no significant coronary artery disease; PCI: percutaneous coronary intervention

Variable	Overall (n = 38)	CAD+ (n = 30)	CAD- (n = 8)	p-Value	Statistical test
Age, years (mean ± SD)	64.4 ± 8.5	65.4 ± 8.2	60.5 ± 9.1	0.146	Mann-Whitney U = 161.0
Male sex, n (%)	29 (76.3)	23 (76.7)	6 (75.0)	1	OR = 1.10 (95% CI 0.18-6.69)
Hypertension, n (%)	29 (76.3)	23 (76.7)	6 (75.0)	1	OR = 1.10 (95% CI 0.18-6.69)
Diabetes, n (%)	27 (71.1)	22 (73.3)	5 (62.5)	0.667	OR = 1.10 (95% CI 0.18-6.69)
Dyslipidemia, n (%)	24 (63.2)	21 (70.0)	3 (37.5)	0.117	OR = 3.89 (95% CI 0.76-19.86)
Smoking, n (%)	17 (44.7)	14 (46.7)	3 (37.5)	0.709	OR = 1.46 (95% CI 0.29-7.23)
Chronic kidney disease, n (%)	6 (15.8)	5 (16.7)	1 (12.5)	1	OR = 1.40 (95% CI 0.14-14.03)
History of CAD, n (%)	14 (36.8)	13 (43.3)	1 (12.5)	0.216	OR = 5.35 (95% CI 0.58-49.10)
Prior myocardial infarction, n (%)	10 (26.3)	10 (33.3)	0 (0.0)	0.082	OR = 8.71 (95% CI 0.46-165.96)
Prior PCI/stent, n (%)	13 (34.2)	12 (40.0)	1 (12.5)	0.222	OR = 4.67 (95% CI 0.51-42.92)
Typical angina as a presenting symptom, n (%)	27 (71.1)	22 (73.3)	5 (62.5)	0.667	OR = 1.65 (95% CI 0.32-8.54)

None of the demographic variables, cardiovascular risk factors (including chronic kidney disease), or history of coronary disease differed significantly between positive and negative CAD patients, although prior myocardial infarction showed a nonsignificant trend toward being more frequent in the positive CAD group (33.3% vs. 0%; OR 8.71, 95% CI 0.46-165.96; p = 0.082).

PTP of CAD

PTP of obstructive CAD was calculated using the ESC 2024 risk factor-weighted clinical likelihood model. Across the entire cohort, the distribution of PTP categories showed a large predominance of moderate probabilities: 68.4% of patients (26/38) had a PTP between 15% and 50%. Low probabilities accounted for 21.1% of cases (8/38), while very low probabilities (<5%) were found in 10.5% of patients (4/38). No patients were classified in the high PTP category (>50%).

To evaluate whether PTP discriminated between patients with and without angiographic CAD, PTP categories were grouped into two strata. Their distribution according to angiographic status is shown in Table 2.

**Table 2:** ESC 2024 PTP strata according to angiographic status Fisher’s exact test: p = 0.689 CAD+: significant coronary artery disease; CAD-: no significant coronary artery disease; ESC: European Society of Cardiology; PTP: pretest probability

PTP group	CAD+ (n = 30)	CAD- (n = 8)
Low/very low PTP	9	3
Moderate/high PTP	21	5

There was no significant difference in the proportion of low/very-low versus moderate PTP between CAD+ and CAD- patients (OR 1.40, 95% CI 0.27-7.15; Fisher’s exact p = 0.689). Among patients classified as very low or low PTP (n = 12), 10 were diabetic (83.3%).

DSE versus angiographic findings

The mean number of ischemic segments identified on DSE in the overall cohort was 3.17, with a median of 3 segments. When patients were stratified according to coronary angiography results, a clear distinction emerged between the two groups. Patients with CAD+ (n = 30) had an average of 3.64 ischemic segments, while those whose coronary angiography revealed no significant lesions (CAD-, n = 8) had a significantly lower average number, limited to 1.50 segments. The difference in ischemic burden between CAD+ and CAD- patients was statistically significant (Mann-Whitney U = 181.0; p = 0.008).

Territorial concordance between DSE and coronary angiography, defined as agreement between the ischemic territory on DSE and the corresponding epicardial vessel according to the standard myocardial perfusion territories described in the ESC 2024 CCS guidelines, was observed in 67.6% of patients.

The correlation between the number of ischemic segments on DSE and the number of significantly diseased epicardial vessels on angiography was modest, with a Pearson correlation coefficient of r = 0.19 (t = 1.15; p = 0.26).

Number of diseased vessels and treatment strategy

The extent of epicardial disease and the corresponding therapeutic strategy are summarized in Table 3.

**Table 3:** Number of significantly diseased vessels and therapeutic strategy CABG: coronary artery bypass graft; PCI: percutaneous coronary intervention

Number of significantly diseased vessels	Medical therapy (n)	PCI (n)	CABG (n)	Total (n)
Zero vessels	8	0	0	8
One vessel	0	23	0	23
Two vessels	0	2	1	3
Three vessels or more	0	0	4	4
Total	8	25	5	38

Overall, 30 out of 38 patients (79.9%) underwent revascularization (25 PCI and five CABG), whereas eight patients (18.4%) with no significant stenosis were managed with optimal medical therapy alone. Single-vessel disease was exclusively treated with PCI, while patients with more extensive multivessel disease (≥2 vessels) or left main involvement were preferentially referred for CABG.

## Discussion

DSE remains a practical, widely available, and guideline-supported tool for assessing myocardial ischemia in CCSs. Recent ESC recommendations highlight its value as a first-line functional imaging test in patients with moderate or high likelihood of obstructive CAD [1], particularly in settings where access to advanced imaging is limited. Real-world data from different regions, including resource-constrained environments, have also confirmed its good diagnostic performance and favorable safety profile, supporting its continued use as a gatekeeper to invasive angiography [4,5].

In our population, territorial concordance between the ischemic territory identified on DSE and the corresponding epicardial vessel on angiography reached approximately two-thirds. This level of agreement is consistent with prior work comparing stress echocardiography and invasive coronary anatomy. For example, Jang et al. reported similar concordance in patients undergoing treadmill stress echocardiography when ischemic territories were assigned to standard coronary distributions [6]. Earlier studies comparing DSE, nuclear perfusion imaging, and coronary angiography also described only moderate agreement in mapping the extent and localization of CAD [7].

Functional-anatomical discordance is well recognized: several investigations have demonstrated limited concordance between stress-induced abnormalities and intracoronary fractional flow reserve (FFR), both for the presence and localization of ischemia [8,9]. Coronary microvascular dysfunction, particularly prevalent among diabetics, likely contributes to this discrepancy. Contemporary reviews highlight that microvascular disease is common, can generate ischemia in the absence of significant epicardial stenoses, and may alter the relationship between regional wall-motion abnormalities and angiographic findings [10-12]. Taken together, these data confirm that the ≈68% concordance observed in our series is aligned with published evidence and highlight that DSE offers complementary functional information rather than a simple anatomical map.

European guidelines emphasize the importance of estimating the PTP of obstructive CAD to avoid unnecessary invasive investigations [1]. However, the complementary challenge is to avoid the opposite pitfall, underestimating the likelihood of coronary disease and thereby missing clinically significant CAD. Several studies have therefore evaluated how different PTP models perform in real-world settings. One analysis based on the 2019 ESC framework showed that revisions to PTP definitions affected the diagnostic accuracy of DSE and that substantial overlap in risk profiles limited discrimination, particularly in high-risk cohorts with a heavy burden of diabetes [13]. Although the 2024 ESC revision refined this approach by integrating the number of cardiovascular risk factors into the risk-factor weighted clinical likelihood (RF-CL) [1], our findings show that very-low and low PTP categories still underestimated the true prevalence of obstructive CAD in our population.

These observations echo the concerns raised in recent commentaries on the CCS 2024 guidelines, which stress that RF-CL, despite providing a more realistic estimate at the population level, must be complemented by additional clinical and imaging criteria to avoid missing or underdiagnosing CCS [14]. This raises an important practical question: when should clinicians rely on PTP alone, and when should they adopt a more vigilant approach?

Our findings suggest that, in such environments, imaging tests like DSE remain crucial to refine risk beyond clinical likelihood estimates, especially given that DSE is noninvasive, radiation-free, and considerably more affordable than other advanced imaging modalities [15,16]. This is particularly relevant in regions such as North Africa and the Middle East, where the high prevalence of diabetes, hypertension, and dyslipidemia significantly influences the performance of PTP models. Epidemiological evidence from the Global Burden of Disease program and regional studies confirms that metabolic risk factors and ischemic heart disease represent a major and growing health burden in these countries, including Morocco [17,18]. World Health Organization cardiovascular risk charts similarly underline the need for accessible, cost-effective diagnostic strategies in these settings [19].

In therapeutic terms, nearly 80% of patients undergoing coronary angiography after a positive DSE required revascularization, mostly by PCI, with CABG reserved for more extensive disease. This substantial rate of downstream intervention highlights the important therapeutic implications of DSE beyond its diagnostic role. Contemporary data show that abnormal stress echocardiography is strongly associated with obstructive CAD and future adverse events and that these findings often prompt intensification of preventive therapy or invasive management [5]. Furthermore, the significantly higher number of ischemic segments observed in patients with angiographically confirmed CAD supports the concept that the extent of stress-induced wall-motion abnormality broadly reflects ischemic burden, as suggested in smaller cohorts evaluating DSE positivity in relation to the extent of coronary disease [15,20]. In contrast, the mean number of ischemic segments among CAD-negative patients in our study was low (1.50), which is consistent with the fact that several of these tests were considered positive not because of a large ischemic burden but due to typical anginal symptoms and stress-induced ECG changes rather than marked wall-motion abnormalities.

Only a limited number of studies have directly compared the number of ischemic segments on DSE with the number of significantly diseased vessels on angiography, making our analysis relatively original in this regard. In our cohort, the correlation between these two variables was modest (r ≈ 0.19). This observation should be interpreted with caution, as the small sample size limits statistical power and the presence of symptom- or ECG-driven DSE positivity naturally attenuates linear associations between functional and anatomical markers of disease. Despite these limitations, our findings still reinforce the value of DSE as an integrative tool for risk stratification and therapeutic decision-making in CCSs.

Patterns of revascularization in our cohort were consistent with contemporary guideline-based practice. Single-vessel disease was treated exclusively with PCI, whereas multivessel or left main disease more frequently led to referral for CABG. These decisions align with the 2018 ESC/EACTS Guidelines on Myocardial Revascularization and their subsequent joint review, which recommend CABG as the preferred strategy for complex three-vessel or left main disease, while considering PCI appropriate for less complex anatomy or when surgical risk is elevated [2,21]. Long-term data from the SYNTAX Extended Survival (SYNTAXES) study further support a survival advantage of CABG in patients with three-vessel disease [22], and findings from the FAME 3 trial showed that FFR-guided PCI did not achieve noninferiority compared with CABG in complex multivessel disease [23,24]. The consistency of our therapeutic decisions with these evidence-based recommendations strengthens the external validity of our results and suggests that DSE-guided management was embedded within an appropriate revascularization framework.

This study has several limitations. It is retrospective, monocentric, and based on a relatively small sample. Only patients with positive or equivocal DSE referred to angiography were included, introducing selection bias and preventing accurate estimation of specificity or negative predictive value. Furthermore, the absence of long-term clinical follow-up limits assessment of the prognostic impact of revascularization in this population.

Despite these limitations, our study illustrates how DSE can meaningfully influence therapeutic decision-making in patients with CCSs in a real-world Moroccan setting. Future research should validate these findings in larger multicenter cohorts, compare DSE with coronary CT angiography and stress CMR across diverse clinical scenarios, explore the added value of quantitative and AI-based analysis of stress-induced wall-motion abnormalities, and assess cost-effectiveness and long-term outcomes of DSE-guided strategies in middle-income health systems.

## Conclusions

In this real-world cohort of patients with CCSs referred for coronary angiography after a positive or equivocal DSE, we found a high prevalence of significant CAD and a substantial rate of revascularization. The extent and localization of inducible ischemia on DSE were strongly aligned with angiographic anatomy and closely guided the choice between PCI and CABG, with PCI predominating in single-vessel disease and CABG reserved for more complex multivessel or left main involvement.

The observation that several patients with low or very low PTP, most of whom were diabetic, ultimately had significant CAD underscores the limitations of clinical likelihood models in high-risk metabolic populations and highlights the continued importance of functional assessment. Taken together, these findings reinforce the role of DSE as a practical, accessible, and clinically meaningful gatekeeper to invasive evaluation and revascularization in CCS, fully consistent with contemporary ESC CCS and ESC/EACTS revascularization guidance.
